# Extensive Visual Training in Adulthood Reduces an Implicit Neural Marker of the Face Inversion Effect

**DOI:** 10.3390/brainsci14020146

**Published:** 2024-01-30

**Authors:** Simen Hagen, Renaud Laguesse, Bruno Rossion

**Affiliations:** 1 Université de Lorraine, CNRS, IMoPA, F-54000 Nancy, France; simen.hagen@univ-lorraine.fr; 2 Psychological Sciences Research Institute, UCLouvain, 1348 Louvain-La-Neuve, Belgium; renaud.laguesse@uclouvain.be; 3 Université de Lorraine, CHRU-Nancy, Service de Neurologie, F-54000 Nancy, France; 4 Université de Lorraine, CHRU-Nancy, Service de Neurochirurgie, F-54000 Nancy, France

**Keywords:** face identify recognition, face inversion, neural plasticity, frequency tagging, EEG

## Abstract

Face identity recognition (FIR) in humans is supported by specialized neural processes whose function is spectacularly impaired when simply turning a face upside-down: the face inversion effect (FIE). While the FIE appears to have a slow developmental course, little is known about the plasticity of the neural processes involved in this effect—and in FIR in general—at adulthood. Here, we investigate whether extensive training (2 weeks, ~16 h) in young human adults discriminating a large set of unfamiliar inverted faces can reduce an implicit neural marker of the FIE for a set of entirely novel faces. In all, 28 adult observers were trained to individuate 30 inverted face identities presented under different depth-rotated views. Following training, we replicate previous behavioral reports of a significant reduction (56% relative accuracy rate) in the behavioral FIE as measured with a challenging four-alternative delayed-match-to-sample task for individual faces across depth-rotated views. Most importantly, using EEG together with a validated frequency tagging approach to isolate a neural index of FIR, we observe the same substantial (56%) reduction in the neural FIE at the expected occipito-temporal channels. The reduction in the neural FIE correlates with the reduction in the behavioral FIE at the individual participant level. Overall, we provide novel evidence suggesting a substantial degree of plasticity in processes that are key for face identity recognition in the adult human brain.

## 1. Introduction

Human faces constitute a special visual category from which we rapidly identify people based on their idiosyncratic facial characteristics—face identity recognition (FIR). Human adults‘ performance at this socially crucial function is impressive, with thousands of faces to recognize individually [1], rapidly (e.g., within 1–2 fixations and a few hundreds of milliseconds; [2,3,4]), and automatically (i.e., without the intention to do so and being able to suppress it; [5,6]). In neurotypical human adults, simply flipping a face upside-down drastically impairs FIR, even though upright and inverted faces contain exactly the same visual content (the face inversion effect [FIE]; [7,8]). The FIE is weak or even non-existent in other animal species including non-human primates [9,10,11], and despite more than 50 years of investigation, it is still debated what factors give rise to the increased sensitivity to upright faces in humans. As detailed below, current evidence pointing toward both biological constraints at birth and visual experience accrued over development. However, the extent to which this specialization is malleable after development, in the mature adult brain, is less known and a topic of the current study.

Several lines of evidence support the role of biological constraints present in the visual system at birth (for review, see [12]). For example, human newborns preferentially orient to faces or face-like stimuli relative to inverted faces and other visual controls [13,14], even though this preference seems to be driven by more general constraints than faceness per se [15]. Consistent with this, EEG responses measured in 1- to 4-day-old awake human newborns show stronger signal to upright than inverted schematic face-like patterns [16]. Also, a recent study testing a person born with a condition that causes his head to be rotated back showed that his FIR performance for upright faces was as poor as for inverted faces, which matched the inverted face discrimination of controls, suggesting that lifetime experience with inverted faces was not sufficient to overcome initial biological constraints [17].

However, a wealth of evidence indicates that visual experience accrued after birth plays a critical role in shaping FIR and the FIE in particular. For example, people who lack visual input to the retina from birth until 2–18 months after birth, due to dense congenital bilateral cataracts, show a persistent FIR deficit in later adult age [18,19,20,21], to the extent that upright FIR is nearly as poor as for inverted faces [19], and the deficit is especially prominent if the lack of visual input is to the right hemisphere [20]. More generally, and although there are discrepancies between studies, the FIE emerges relatively late in development and increases progressively until adulthood [22,23,24]. While even a late emergence and gradual increase does not rule out the contribution of genetic factors, there is independent evidence that the FIR system remains plastic until at least in late childhood. For example, the ORE (i.e., better FIR for own than other “race” faces; [25,26,27]: see [28] for a recent special issue on this other-race face effect), which may start to develop by the age of 9 months [29,30], is reversed for adult Asians who are exposed to Caucasian faces in late childhood following adoption by western European families [31,32]. Thus, visual experience during development plays a critical role in tuning the system to allow for the rapid and automatic FIR characteristic of the mature system.

A growing body of evidence also suggests that the FIR system, in the adult mature brain, remains plastic. For example, face individuation training with other-race faces attenuates the behavioral ORE [33,34,35], while training with other-age faces attenuate the behavioral other-age effect [36,37,38]. However, the strongest evidence arguably comes from studies examining whether individuation training of inverted faces can influence the FIE [39,40,41,42]. While early studies did not test whether inverted training generalized to inverted face identities not seen during training [39,42], or failed to find such a generalization effect [40], Laguesse et al. [41] employed an extensive inverted face discrimination training protocol—that mitigated downfalls in earlier studies—and found that it led to a robust improvement in inverted FIR that generalized to novel faces, thereby leading to a substantial reduction (40%) in the FIE. Thus, behavioral evidence now suggests that the FIR system may still be highly plastic well after maturation.

**Figure 1:**
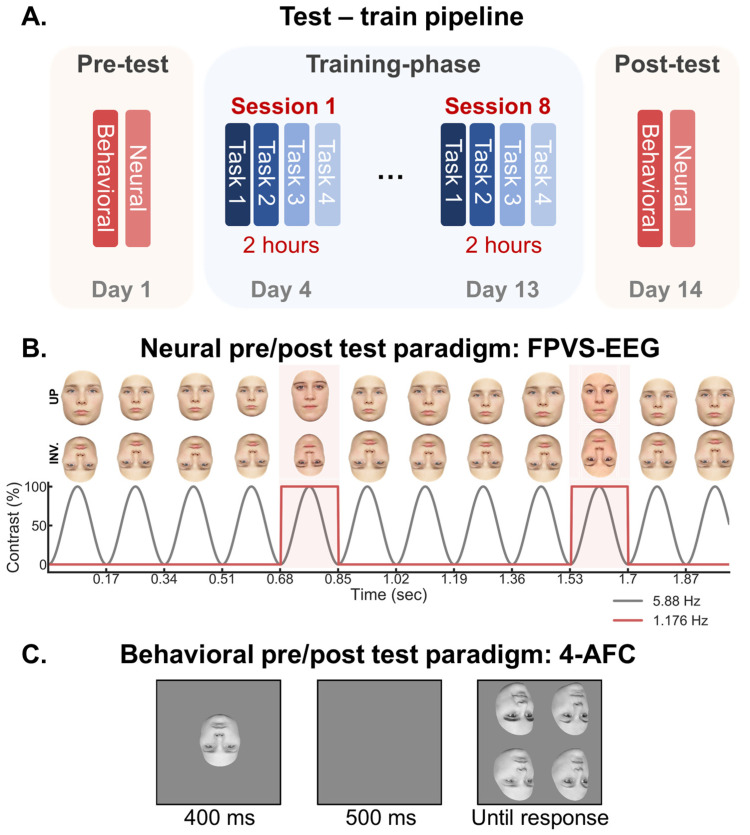
(**A**) Schematic illustration of the test and training pipeline, where eight training sessions, with up to four different training tasks per session (see Table 1), were flanked by pre- and post-training tests. The pre- and post-training tests consisted of separate neural and behavioral measures, as illustrated in the next panels. (**B**) Schematic illustration of the FPVS-EEG experimental paradigm. Separate sequences showed upright and inverted faces. The test was administered before and after training. The participants fixated a cross centered between the eyes and pressed the space bar every time they detected random changes in color from blue to red. (**C**) The four-alternatives forced-choice (4-AFC) delayed matching task [41], which was performed for both upright and inverted faces before and after training (different sets of faces).

However, with behavioral evidence obtained in explicit tasks only, an outstanding issue is whether this modulation of the FIE truly reflects the core FIR function or a change in other general cognitive systems (e.g., attention, decision making), or even include task-specific learning. We address the issue in the present study by replicating the previous behavioral study that successfully trained inverted face individuation [41], (critically) adding a robust and valid neural measure of FIR with EEG before and after training. Specifically, the participants underwent extensive training (2 weeks, ~16 h) discriminating a set of 30 unfamiliar inverted faces across viewpoints and in different learning tasks (Figure 1A; Table 1). Pre- and post-training, they completed a behavioral FIR test where they matched a study face to one out of four test faces presented in a different depth-rotated view (Figure 1C; [41]). Crucially, unlike previous studies, the pre- and post-tests also included a separate neural measure of FIR, namely, EEG coupled with a validated frequency tagging approach (Fast Periodic Visual Stimulation [FPVS]-EEG) for isolating automatic FIR neural responses (Figure 1B; [5]).

In this paradigm, an unfamiliar face identity is repeated at a fixed frequency rate (*F,* usually 6 Hz) for about one minute, with different face image identities interleaved as every fifth item (*F*/5; i.e., 1.2 Hz), with image size varying at each cycle to minimize low-level repetition effects (Figure 1B). Thus, a face individuation signal is objectively quantified at the F/5 frequency (1.2 Hz) and associated harmonics (2.4 Hz, 3.6 Hz, etc.; [5]; for review: see [43]). This approach provides an objective identification of FIR responses (i.e., at experimentally defined frequencies) and full quantification (as a sum of harmonics) of neural activity in the EEG frequency domain. Previous research has shown a robust FIE in this paradigm [5] (53% of signal decrease with inversion: see [43]) and validated its effectiveness at isolating neural FIR mechanisms. For instance, there is no FIR signal in the well-known case of prosopagnosia PS [44], and a substantial reduction in cases of developmental prosopagnosia/prosopdysgnosia [45]. Moreover, when coupled with spatially resolved intracerebral EEG, the paradigm measures strong FIR signals in ventral occipo-temporal cortex (VOTC) regions considered core face to this function, i.e., the inferior occipital and fusiform gyri with a right hemispheric dominance [46]. Finally, within these regions, amplitude maxima in this paradigm have been identified at the very same intracerebral electrode contacts, leading to transient and specific FIR impairments in several cases [47].

Here, with measures on the scalp (EEG), we hypothesized that if inverted face training modulates these neural face FIR processes, then there would be an increase in the FIR amplitude at *F*/5 frequencies and harmonics for inverted novel faces.

## 2. Methods

Participants. Twenty-eight participants (ten males; mean age = 22.9 years, SD = 1.75 years) took part in the study, a sample that is sufficiently large to measure the FIE with FPVS-EEG [5] and to perform reliable correlation analyses between behavioral and neural measures. All participants were native French speakers with normal or corrected-to-normal vision. The participants were informed about the training and testing aspects of the experiment and that they could opt out at any time throughout the experiment. Subsequently, participant consent was obtained from each participant. Two out of the twenty-eight participants were excluded from the analysis due to inconsistent scalp topographies and lack of ROIs (see Section 2.3 below). The study was approved by the human ethical committee of UCLouvain and the participants received monetary compensation for their participation.

Stimuli. The stimuli in the behavioral tasks (training and pre- and post-tests) were identical to that used in a previous study [41] and consisted of a total of 118 high-quality color photographs of face identities that varied across three viewpoints: (1) full-front, (2) 30 degrees depth rotation to the right (3) and to the left (Figure 1). A subset of these images (*n* = 64) was used to test for inversion effects in the pre- (*n* = 32; 16 females, 16 males) and post- (*n* = 32, 24 females; 8 males) training tests. All face images were cropped from their background, contained no external features (e.g., hair), and were pasted on a uniform gray background. The images ranged in size from 170 to 200 pixels wide and 250 pixels height. The faces were flipped vertically for the inverted condition.

The stimuli in the FPVS-EEG pre- and post-test were identical to that used in previous studies with this paradigm [5] and consisted of full-front colored photographs of 25 male and 25 female faces with a neutral expression. They were taken under standardized conditions in terms of background, lighting, and distance from the camera. Moreover, external features such as hair and ears were cropped out using Adobe Photoshop before the isolated faces were pasted against a gray background. Final images were resized to a height of 250 pixels (width = 186 ± 11 pixels). The faces were flipped vertically for the inverted condition. The mean luminance of the images was equalized online during stimulation and a gamma correction was applied.

Apparatus. The different tasks were run using the Psychtoolbox (MATLAB; Mathworks, Inc., Natick, MA, USA), E-Prime (Psychology Software Tools, Pittsburgh, PA, USA), or JavaScript. For the behavioral tasks, the participants were seated in a dimly lit room approximately 70 cm from the computer screen. For the EEG recording, the participants were seated in a dimly lit and sound-attenuated room approximately 100 cm from the computer monitor (screen resolution of 800 × 600 pixels with a frame rate of 100 Hz).

The EEG was acquired at 512 Hz using a 128-channel Biosemi Active II system (Biosemi, Amsterdam, Netherlands), with electrodes including standard 10–20 system locations as well as additional intermediate positions. Two additional electrodes (Common 252 Mode Sense [CMS] active electrode and Driven Right Leg [DRL] passive electrode) were used as reference and ground electrodes, respectively. Eye movements were monitored using four electrodes placed at the outer canthi of the eyes and above and below the right eye. In both the behavioral and the EEG paradigms, the images subtended approximately 4.0 horizontal and 6.53 vertical degrees of visual angle. A chinrest was used to avoid movement of the head throughout the experiments.

### 2.1. Training Sessions

Table 1 illustrates the training protocols of the study, which were identical to the protocol used by Laguesse et al. [41]. The participants were trained to individuate thirty inverted faces, split across three face sets with each set of ten faces. The face sets were progressively introduced over the course of a two-week period and across four different tasks: exposure/learning, naming, old/new recognition, and forced-choice matching, all of which are described below and elsewhere [41]. Each participant underwent individual training of approximately 2 h per day for 8 days, for a total of approximately 16 h of training. The sessions took place in late afternoon or in early evening to facilitate the consolidation of learning [48].

Exposure/learning task. The three face sets were presented separately in three identical tasks (E1, E2, E3) and each task was divided into six blocks. In the first block, the participants were exposed to five inverted full-front face pictures together with their names. Each inverted face-and-name combination was presented twice. The participants were instructed to associate the name with the face. In the second block, the same five faces were presented but in the other two depth-rotated views (30L, 30R). Each face was presented separately for 5 s. In the third block, the participants were first presented with a name-list of the already exposed faces (without their faces). Next, the faces were presented separately (without labels) and the participants were instructed to name the faces. The images were shown twice in each of the three viewpoints (10 faces × 3 viewpoints = 30 trials). The same procedure was used in the three subsequent blocks for the other five faces of the set.

Naming task. The three face sets were presented separately in three identical tasks (N1, N2, N3). Before a task, the participants were provided with a list of the relevant face names. After seeing the list, the participants performed the task, in which the faces were presented separately on the screen until the participant pressed the keyboard key corresponding with the first letter of the face name (once the third face set was introduced, the participants also provided verbally the full name of each face). A visual feedback that contained the accuracy of their keypress (‘‘correct’’/‘‘incorrect’’) and the correct name was provided after each key press. Each task contained a total of 30 trials (10 faces × 3 viewpoints). The task repeated until an accuracy of 100% was reached. The list with the names of the faces was provided before starting the task each time.

Once the participants had completed N1 and N2, they were combined into one test (N1 + N2 = 20 trials; for overview of training protocol: see Table 1). Moreover, once the participants had completed N3, all three tests were combined into one (N1 + N2 + N3 = 30 trials).

Old/New task. An old/new recognition task was administered subsequent to the exposure/learning and naming tasks (three old/new tasks in total). In a trial, a face image was shown from one of three possible viewpoints (FF, 30L, 30R) and the participant had to indicate with a keypress if the face was previously seen (old) or novel (new). The first task contained face set 1 (old) and face set 2 (new; 20 faces × 3 viewpoints = 60 trials). The second task contained face set 1 and 2 (old) and face set 3 together with an additional 10 novel faces (new; 40 faces × 3 viewpoints = 120 trials). The third task consisted of face sets 1, 2, and 3 (old) and 30 novel faces (new; 60 faces × 3 viewpoints = 180 trials).

4AFC Delayed Matching Task during the Training Session. In a given trial, a face was presented for 400 ms, followed by a brief delay (500 ms) and then by four faces arranged in a square around the center of the screen, which remained visible until the participant’s response. The task was to indicate with a key press which one of the four faces was presented at encoding. The participant received visual feedback after each response about the accuracy of the trial and the overall accuracy at that point in the task. The faces were always presented in the inverted (INV) orientation.

The first (target) face was always presented in a different depth-rotated view than the four subsequent faces, which were all presented in the same depth-rotated view. Six depth-rotated view combinations between the target and the distractor faces were possible: Full-front (FF)-30 Right (R); 30R-FF; FF-30 Left (L); 30L-FF; 30L-30R; 30R-30L. The test consisted of a total of 56 faces, 32 of which were present at pre-test and 24 new faces. There were a total of 672 possible trials (56 faces × 6 depth combinations × 2 repetitions), out of which 336 were randomly selected for each participant and session.

Responses were made with the two hands using the keyboard keys 1, 3, 4, and 6 on the numeric pad, corresponding to the four positions on the screen of the four faces to discriminate (see Figure 1C).

### 2.2. Testing Sessions

The participants were tested in two sessions: a pre- and post-training test. The first test occurred 3 days prior to the first training session (Figure 1A). The second test occurred 1 day after the last training session. In both sessions, the participants were tested in a behavioral four-alternative forced-choice test (4AFC), as used previously by Laguesse et al. [41], and in a separate FPVS-EEG protocol, both of which are described in detail below. The order of the behavioral and EEG tests was counterbalanced across the participants. The pre- and post-tests used different images than those used during training, thereby controlling for learning effects that are stimuli specific.

4AFC Delayed Matching Task during the Testing Sessions. The participants completed a pre-and post- 4AFC test (Figure 1C; [41]). This test was similar to the 4AFC training paradigm, with the exception that the (a) face images were all novel (32 in each test), (b) the images were shown randomly in both upright (UP) and inverted (INV) orientations (target and distractor faces were always in same orientation), and (c) no feedback was provided after each trial. There were a total of 384 possible trials (32 faces × 6 depth combinations × 2 orientations) out of which 280 were randomly selected for each participant and session to limit the duration of testing. The participants were instructed to respond as rapidly as possible while maintaining high levels of accuracy.

EEG Recording during Testing Sessions. The pre- and post- FPVS-EEG test consisted of presenting face images periodically for 64 s during EEG recording, with upright and inverted faces presented in separate sequences (Figure 1B). In a given sequence, a frontal-view face image was presented at a base rate of 5.88 cycles per second (5.88 Hz = base stimulation frequency) through sinusoidal contrast modulation using a custom MATLAB script [5]. The images varied randomly in size (between 80% and 120% in 2% steps) at every cycle of presentation to prevent adaptation in low-level retinotopic areas. Each sequence started with a fixation-cross displayed for a random duration of 2 to 5 s, followed by a 2 s fade-in during which the image stimulation progressed gradually from 0% to 100% contrast level. This was to avoid eye movements at the beginning and end of the stimulation. Within each sequence, a face image was randomly selected to serve as a base image (e.g., Face A) that was presented repeatedly for four consecutive cycles (each cycle = 170.07 ms), and at every fifth cycle (5.88/5 = 1.176 Hz) different face images (e.g., Face B, C, D) presented in place of the base face. This manipulation ensures that a signal occurring at 1.176 Hz reflects a differential signal between the base and the oddball faces, which is an indication that the neural system is discriminating between the individual faces despite variations in image size.

During image presentation, the participants performed an orthogonal task where they were instructed to fixate a cross—centered between the eyes of the faces—and to press the space bar every time they detected random changes in color from blue to red (duration of color change: 200 ms; six changes randomly timed in each sequence). This task ensured that the participants would maintain attention in the center of the objects while removing explicit task demands to the images.

In total, each FPVS-EEG session consisted of eight 60 s image sequences. Four of these sequences contained upright faces and four contained inverted faces. Moreover, they each comprised two sequences of female faces and two sequences of male faces. Thus, each session consisted of 8 min of *image* stimulation (60s × 4 repetitions × 2 conditions) and short breaks were provided in between each sequence. The order of upright and inverted sequences was random.

### 2.3. EEG Analysis

Preprocessing. Preprocessing was conducted with a customized software (Letswave 5: http://nocions.webnode.com/letswave, accessed on 1 December 2023) running in the MATLAB environment (The Mathworks). EEG data were band-pass filtered (0.1–120 Hz zero-phase Butterworth filter, 24 dB/octave slope). A notch filter was applied to remove 50 Hz line-noise (50, 100 Hz). The data were then down sampled from 512 Hz to 256 Hz, and each stimulation sequence was segmented into epochs starting 2 s before the first image presentation until 2 s after the last image presentation (−2 to 62 s). To isolate and remove large artifacts generated by eye blinks, independent component analysis (ICA) was applied to the data of four and five participants in the pre- and post-test, respectively, who blinked more than 0.20 times/second on average during the sequences (one component removed in each case). All channels were subsequently re-referenced to the common average reference, and the data were cropped down to an integer number of 1.176 Hz that contained the start of the first presentation cycle and the end of the last presentation cycle (0–59.5238 s, 70 oddball cycles, 15,238 time bins). The segmented sequences were averaged within each participant separately for each condition in the time domain to increase signal-to-noise ratio; (e.g., [44]). One segment was excluded from one participant due to a lack of signal in all channels.

Frequency domain analysis. To extract amplitude spectra for all channels, a Fast Fourier Transform was applied to the averaged segments (frequency resolution, 1/59.5238, i.e., 0.017 Hz). To determine the number of harmonics included in the discrimination and general signals, the amplitude data from the frequency domain for each session (session, orientation) were first averaged across the participants and channels. Next, the amplitude in each frequency bin was converted to Z-scores by subtracting its amplitude from the average amplitude of the ten surrounding bins in each direction, excluding the two directly neighboring bins (SNS: signal-to-noise subtraction), and dividing it by the standard deviation of the twenty surrounding bins. Harmonics for the discrimination and general responses were extracted and analyzed until they were no longer significant at a threshold of *z* > 1.96 (*p* < 0.05; i.e., signal > noise; (e.g., [5,44])). This criterion was equal across all conditions (session, orientation). For the discrimination frequency, the harmonics were analyzed up to the seventh harmonic (7 *F*/5 = 9.41 Hz), excluding the fifth harmonic (5 *F*/5 = 5.88 Hz) because it overlaps with the fundamental frequency of the base response (*F* = 5.88 Hz). For the general frequency, the harmonics were analyzed up to the eighth harmonic (8*F* = 47.04 Hz).

For subsequent analysis and to consider noise variations across the EEG spectrum, we converted the amplitude in each frequency bin to a baseline corrected signal-to-noise subtraction measure (SNS) by subtracting out the average amplitude of the ten surrounding frequency bins in each direction, excluding the two directly neighboring bins (noise–noise = 0; signal–noise > 0). Separately for each participant and each condition, the discrimination and general response was quantified as the sum of the significant baseline corrected harmonics, as determined by the step defined above.

The regions of interests based on the electrodes that showed the maximum responses for the discrimination (1.172 Hz) and base (5.88 Hz) responses, averaged across all conditions, were separately determined at the individual participant level. This was performed to account for variability in individual scalp topographies in the face discrimination response. For each participant, two channels with the strongest face discrimination response—one from each hemisphere—were selected to serve as the face discrimination ROI (except for two participants where both electrodes were taken from the same hemisphere, since the other hemisphere showed no distinct signal peak). In all the participants, these electrodes were all located in the lateral occipito-temporal regions. The same approach was applied to determine individual ROIs for the common visual response at the base rate. In all the participants, these electrodes were all located in the medial-occipital region. The dissociation between the scalp topography for the discrimination and base response is consistent with previous studies using the same paradigm; for review, see [43].

## 3. Results

### 3.1. Behavioral Results: Pre/Post 4AFC Delayed Matching Task

**Figure 2:**
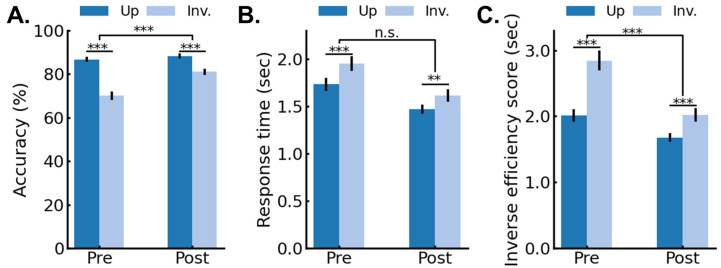
Group behavioral performance as a function of session (pre-, post-) and image orientation (upright, inverted) for (**A**) accuracy, (**B**) correct response times, and (**C**) inverse efficiency scores (RT/proportion correct). Error bars represent standard error of the means. **, ***, n.s. represent *p* < 0.01 *p* < 0.001, *p* > 0.05, respectively.

Accuracy. Figure 2A shows the mean accuracy data (%) for the pre- and post-4AFC test as a function of session (pre-, post-) and image orientation (upright, inverted). The accuracy data were analyzed in a repeated-measures analysis of variance (ANOVA) with session and orientation as within-subjects variables. The significant main effects of session, *F*(1,25) = 49.09, *p* < 0.001, *generalized eta2* = 0.15, indicated that accuracy increased after training. The significant main effect of orientation, *F*(1,25) = 113.13, *p* < 0.001, *generalized eta2* = 0.39, indicated an inversion effect with higher accuracies for upright compared to inverted faces. Crucially, the significant two-way interaction between session and orientation, *F*(1,25) = 30.87, *p* < 0.001, *generalized eta2* = 0.09, indicated that the pre-test FIE (*M_up_* = 86.72%; *SE_up_* = 1.25%; *M_inv_* = 70.04%; *SE_inv_* = 1.94%) was larger than the post-training FIE (*M_up_* = 88.28%; *SE_up_* = 1.21%; *M_inv_* = 81.01%; *SE_inv_* = 1.45%), with a 56% reduction in the FIE in the post-test.

Response time. Figure 2B shows the mean correct response times (RT) for the pre- and post-4AFC test as a function of session (pre-, post-) and image orientation (upright, inverted). RT data were analyzed in a repeated-measures ANOVA with session and orientation as within-subjects variables. The significant main effect of session, *F*(1,25) = 19.43, *p* < 0.001, *generalized eta2* = 0.17, indicated that the RTs were faster after compared to before training. The significant main effect of orientation, *F*(1,25) = 35.33, *p* < 0.001, *generalized eta2 =* 0.07, indicated that the RTs were faster for upright compared to inverted faces. The two-way interaction between session and orientation was not significant, *F*(1,25) = 3.06, *p* = 0.093, *generalized eta2* = 0.003, but it showed a trend in the same direction as the accuracy data (Figure 2B), with a 35% reduction in the FIE in the post-test, ruling out speed-accuracy trade-offs in the pre- to post-sessions.

Inverse efficiency scores. Figure 2C shows the mean inverse efficiency scores (IES; RT/proportion correct) for the pre- and post-4AFC test as a function of session (pre-, post-) and image orientation (upright, inverted). The IES data were submitted to an ANOVA with session and orientation as within-subjects variables. The significant main effect of session, *F*(1,25) = 44.44, *p* < 0.001, *generalized eta2* = 0.22, indicated that the IES were faster in the post- than the pre-test. The significant main effect of orientation, *F*(1,25) = 69.40, *p* < 0.001, *generalized eta2* = 0.23, indicated that the IES were faster for upright compared to inverted faces. The significant two-way interaction between session and orientation, *F*(1,25) = 24.59, *p* < 0.001, *generalized eta2* = 0.05, showed that the pre-test FIE (*M_up_* = 2012 ms; *SE_up_* = 92 ms; *M_inv_* = 2845 ms; *SE_inv_* = 155 ms) was larger than the post-test FIE (*M_up_* = 1680 ms; *SE_up_* = 68 ms; *M_inv_* = 2021 ms; *SE_inv_* = 103 ms), with a 59% reduction in the FIE in the post-test.

### 3.2. EEG Results: Pre/Post FPVS-EEG Test

**Figure 3:**
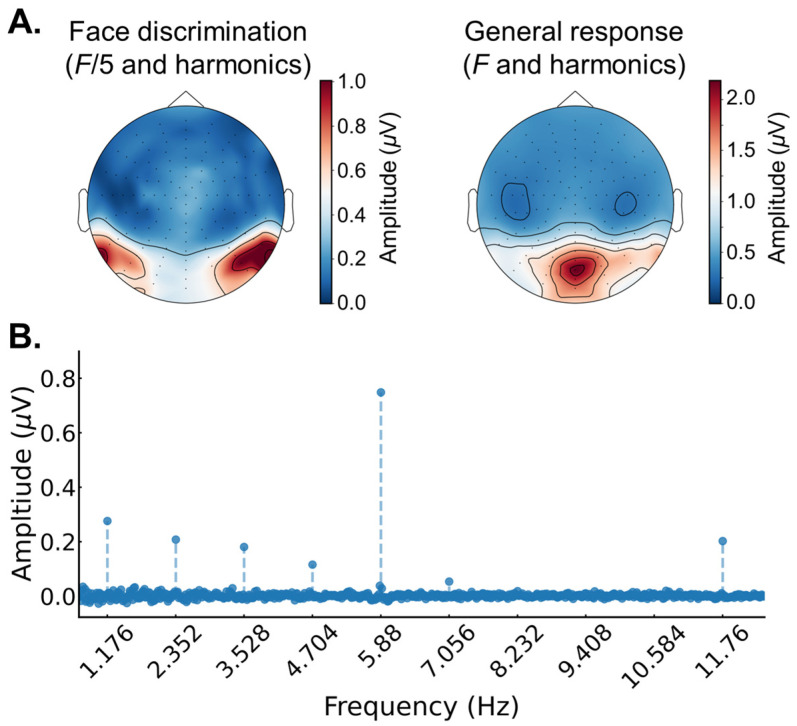
The EEG response averages across conditions (session, image orientation). (**A**) The scalp topography for the face discrimination response (left) and the general visual response (right). (**B**) The EEG amplitude spectrum over the average of two occipito-temporal electrodes (one in each hemisphere: P9, P10). There were clear responses at the face identity discrimination frequency (*F*/5 = 1.176 Hz and its harmonics) and at the general visual stimulation frequency (*F* = 5.88 Hz and its harmonics; only the two first harmonics are shown).

FPVS-EEG responses averaged across all conditions. First, to examine the overall differences between the face identity discrimination (*F*/5 and harmonics) and base (*F* and harmonics) frequency tagged signals, we analyzed the EEG responses averaged across all conditions (i.e., average across session and image orientation, separately for each participant; see Methods for description of response quantification in the frequency domain). Figure 3A shows the scalp topographies for the baseline corrected amplitudes for the face discrimination (*F*/5 and harmonics; left) and the general visual response (*F* and harmonics; right) averaged across all conditions (session, image orientation). For the face discrimination signal, there were clear peaks at bilateral occipito-temporal channels, while for the general visual response there was a clear peak at medial-posterior channels. These topographies are consistent with previous studies using the same paradigm [5]; for review of all studies using this paradigm, see [43]. Figure 3B shows the baseline corrected amplitude spectrum averaged over two peak channels (one in each hemisphere, P9 and P10) and across all conditions (session, image orientation). Obvious signal peaks at 1.176 Hz (*F*/5) and harmonics reflect the onset and offset of different identities in the sequence, while the clear signal peaks at 5.88 Hz (*F*) and harmonics reflect the onset and offset of general responses to any stimuli in the sequence (only the first two harmonics are shown in the graph).

**Figure 4:**
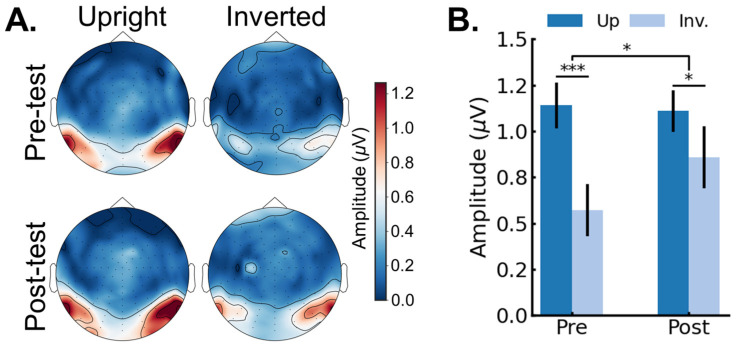
Neural face discrimination responses (*F*/5 and harmonics). (**A**) Scalp topographies displaying the group average face discrimination amplitudes as a function of session (pre-, post-) and image orientation (upright, inverted). (**B**) The average face discrimination amplitude averaged across peak channels as a function of session (pre-, post-) and image orientation (upright, inverted). Error bars represent standard error of the means. *, *** represent *p* < 0.05, *p* < 0.001, respectively.

Identity recognition response. Next, we analyzed whether the face identity discrimination response was influenced by training session (pre-, post-) and image orientation (up, inverted) (see Methods for description of response quantification in the frequency domain). Figure 4A shows the scalp topographies for the group average face discrimination amplitudes (*F*/5 and harmonics) as a function of session (pre-, post-) and image orientation (upright, inverted). Figure 4B shows the face identity discrimination response averaged across peak channels as a function of session and image orientation. The data were analyzed in a repeated-measures ANOVA with session (pre-, post-) and orientation (upright, inverted) as within-subjects variables. The main effect of session was not significant, *F*(1,25) = 2.61, *p* = 0.187, *generalized eta2* = 0.01. The main effect of orientation was significant, *F*(1,25) = 13.22, *p* = 0.001, *generalized eta2* = 0.08, indicating a larger amplitude for upright (*M* = 1.12 µV; *SE* = 0.15 µV) compared to inverted faces (*M* = 0.72 µV; *SE* = 0.12 µV). Crucially, the significant two-way interaction between session and orientation, *F*(1,25) = 6.78, *p* = 0.015, *generalized eta2* = 0.01, indicated that the pre-test FIE (*M_up_* = 1.14 µV; *SE_up_* = 0.17 µV; *M_inv_* = 0.57 µV; *SE_inv_* = 0.11 µV) was larger compared to the post-test FIE (*M_up_* = 1.11 µV; *SE_up_* = 0.14 µV; *M_inv_* = 0.86 µV; *SE_inv_* = 0.12 µV), with a 56% reduction in the FIE in the post-test.

**Figure 5:**
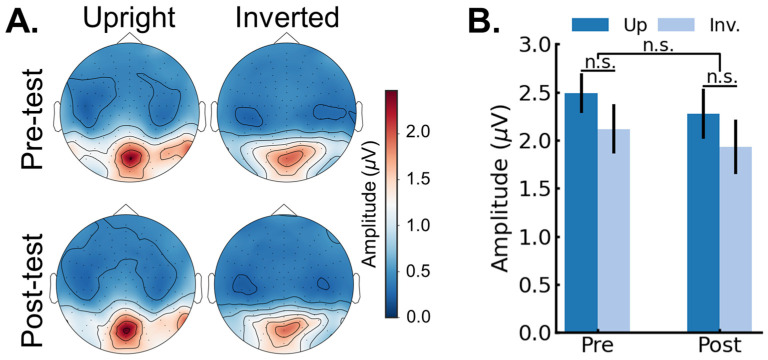
Neural general visual responses (*F* and harmonics). (**A**) Scalp topographies displaying the group average general visual response amplitudes as a function of session (pre-, post-) and image orientation (upright, inverted). (**B**) The average general visual response amplitude averaged across peak channels as a function of session (pre-, post-) and image orientation (upright, inverted). Error bars represent standard error of the means. n.s. represents *p* > 0.05.

General visual stimulation response. As a control analysis, we analyzed whether the general visual response was influenced by training session (pre-, post-) and image orientation (up, inverted). This can serve as a control analysis because this signal captures responses that are evoked by any visual stimuli and that generalize across all faces (i.e., note specific to face identity recognition). Moreover, general visual responses that are frequency tagged at a base frequency capture attentional effects [49]. Thus, training effects that are not specific to face identity recognition should be captured at the general visual response frequency tagged at 6 Hz and harmonics. Figure 5A shows the scalp topographies for the group average general visual response amplitudes (*F* and harmonics) as a function of session (pre-, post-) and image orientation (upright, inverted). Figure 5B shows the general visual response averaged across peak channels as a function of session and image orientation. The data were analyzed in repeated-measures ANOVA with session (pre-, post-) and orientation (upright, inverted) as within-subjects variables. The main effect of the session approached significance, *F*(1,25) = 3.60, *p* = 0.069, *generalized eta2* = 0.006, but this trend was driven by a higher amplitude in the pre- (*M* = 2.30 µV; *SE* = 0.27 µV) compared to the post-session (*M* = 2.10 µV; *SE* = 0.23 µV). The main effect of the orientation approached significance, *F*(1,25) = 3.97, *p* = 0.057, *generalized eta2* = 0.02, indicating a larger amplitude for upright (*M* = 2.38 µV; *SE* = 0.27 µV) compared to inverted faces (*M* = 2.02 µV; *SE* = 0.23 µV). Crucially, the two-way interaction between the session and the orientation was not significant, *F*(1,25) = 0.078, *p* = 0.782, *generalized eta2* = 0.0003.

Correlation between behavior and EEG. The previous analysis showed that the training reduced both the behavioral and neural FIE by increasing the behavioral accuracy and neural amplitude for the inverted FIR. Next, we examined the relationship between the change in the FIE for the behavioral accuracy measure and the neural measure. First, for each participant, we quantified the FIE (upright–inverted), separately for each session. This was performed for both the behavioral accuracy and the neural EEG measure. Second, we quantified a change in the FIE after training, by subtracting the FIE in pre-test from the FIE in the post-test (pre–post). A positive number would indicate a reduction in the FIE, while a negative number would indicate an increase in FIE. Third, we correlated the participant’s behavioral and neural FIE change, using a Pearson correlation test. For the neural measure, this procedure was performed separately for the face identity discrimination signal (*F*/5 and harmonics) and the base rate general signal (*F* and harmonics).

**Figure 6:**
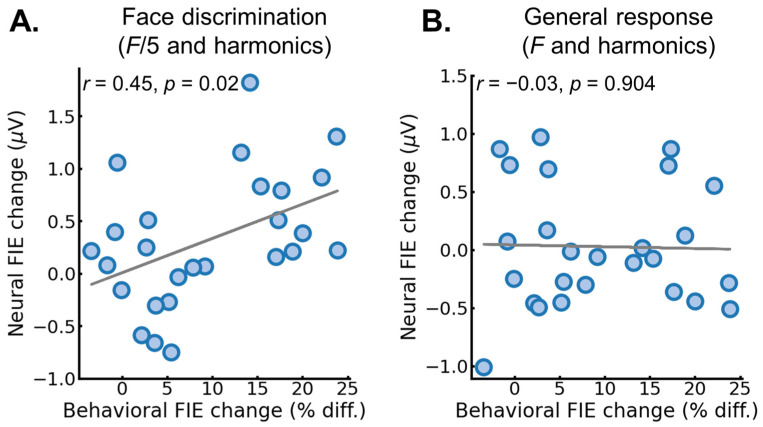
Correlation between behavioral and neural FIE change. (**A**) The correlation between the change in inversion effect from pre- to post-test for the behavioral accuracy measure (*x*-axis) and for the neural face discrimination response (F/5 and harmonics) (*y*-axis). (**B**) The correlation between the change in inversion effect from pre- to post-test for the behavioral accuracy measure (*x*-axis) and for the neural general visual response (F and harmonics) (*y*-axis). A positive change indicates a decrease in the FIE, while a negative change indicates an increase in the FIE. Each colored dot represents an individual participant. Each dot represents a participant and the gray line represents the regression line.

For the discrimination signal (*F*/5 and harmonics), there was a significant positive correlation, *r* = 0.46, *p* = 0.0195, 95% *CI* = [0.08, 0.72], indicating that larger reduction in the neural FIE was associated with a larger reduction in the behavioral FIE (Figure 6A). In contrast, for the base rate general signal (*F* + harmonics), which captures visual signals that are not specific to face identity recognition, there was no significant correlation, *r* = −0.03, *p* = 0.903, 95% *CI* = [−0.41, 0.37] (Figure 6B). Thus, the brain–behavior association was specific to the neural correlates of face identity discrimination.

## 4. Discussion

The goal of this study was to examine if extensive experience, exclusively acquired in adulthood, could influence the rapid and automatic neural processes underlying face identity recognition in humans. Toward that aim, we tested if extensive training with inverted face identity discrimination influenced the discrimination of inverted faces that were not seen during the training. The main finding was that training improved identity discrimination of the novel faces at inverted orientation, as measured both behaviorally (Figure 2) and (most importantly in the present study) neurally (Figure 4), thereby leading to a reduced face inversion effect (FIE). Moreover, we found that larger reductions in the neural FIE were associated with larger reductions in the behavioral FIE (Figure 6A). Finally, control analyses performed on the concurrently measured and isolated general visual responses (6 Hz and harmonics) showed that the training specifically influenced the neural processes involved in face identity recognition and did not extend to general visual processes or attention [49] (Figure 5 and Figure 6B). Overall, our findings provide novel evidence in favor of the claim that FIR processes in the adult mature brain remain highly plastic (e.g., [41]).

Our behavioral findings are consistent with previous behavioral studies, suggesting that the human adult face identity recognition system is influenced by discrimination experience. This previous evidence includes findings that experience can improve discrimination for subclasses of faces for which we do not have saturated experience, including other-race faces (for review, see [27]), other-age faces [36], and inverted faces, (e.g., [41]). The case of inverted face training [41] provides a particularly strong test for experience in adulthood because inverted faces violate the biological fixation constraint at birth, and they are rarely, if at all, experienced during late development. While several studies either failed to find effects of inverted face training that generalized to novel identities not used during training [42], or did not test generalization [39,42], Laguesse et al. [41] reported robust generalization effects following an extensive training protocol that mitigated downfalls of earlier studies. Our replication of their findings, using the same task and a larger participant sample, therefore provides a crucial verification that extensive inverted face identity discrimination training can improve identity recognition for that orientation.

Crucially, our study also extends previous behavioral studies, by examining for the first time whether inverted face training influences the neural processes of FIR. This is important to ascertain because an explicit behavioral measure could reflect training effects on non-perceptual factors, including attentional and decisional factors, and/or learning of task-related strategies. Our findings rule this out, since we observe increased inverted FIR in an implicit neural perceptual discrimination task with novel faces, where the task setup was highly distinct from any of the training tasks. Interestingly, at the group level, the magnitude of the FIE reduction, i.e., 56%, was exactly the same for accuracy rates and the neural measure. Moreover, unlike previous studies that tested whether face individuation training in adulthood with other-race faces [35], or face familiarization through lab experience or real-world encounters [50,51,52], had an effect on a general response to the face (e.g., grouped response to OR-faces before and after training, or familiar versus unfamiliar faces), our neural measure directly isolated the neural processes responding differently to different unfamiliar face identities. While it was recently shown that face identity responses were not modulated by face familiarity (e.g., a few encounters versus extensive encounters; [53]), our findings show that neural FIR responses can depend on discrimination experience, at least when the faces are suboptimally processes by the system. To our knowledge, this is the first evidence that neural FIR processes can be influenced by visual experience exclusively acquired in adulthood.

While we measured global brain activity at the scalp level, and therefore cannot resolve which region(s) instantiated the training effects, it was specific to a few bilateral occipito-temporal electrodes that also showed strong signals during upright face discrimination. Previous studies with EEG/MEG and fMRI have shown that inverted faces are processed by the face recognition system in the VOTC, albeit less efficiently [54,55,56,57,58,59]. Moreover, as presented in the introduction section, previous research on cases of prosopagnosia (due to brain damage or intracerebral electrical stimulation) and intracerebral recordings has validated the effectiveness of the current FPVS-EEG approach at isolating neural FIR processes [44,46,47]. Taken together, this suggests that the increased neural inverted discrimination was instantiated in face-selective regions also involved in discrimination of upright faces. Nevertheless, future studies using more spatially resolved recording methods would need to ultimately verify this conclusion and further test if different face-selective regions exhibit different degrees of plasticity (e.g., adopting our training approach with fMRI).

Previous studies focusing on neural correlates of human face recognition have largely focused on the developmental trajectory of the face-selective system in the VOTC (for recent review: see [60]). Research has shown that face-selectivity in FFA exists already at the age of 2.8 months [61] and that, throughout development, local clustering of face-selective responses increases in at least the inferior-occipital-gyrus and posterior fusiform gyrus [62,63,64,65,66,67,68]. For face individuation responses, an ERP study showed responses in 2.5–5-month olds that were specific to the right hemisphere [69]; also see [24] for 5 year olds. Using fMRI, face individuation responses in face-selective regions has been shown in 5-year olds (youngest group tested; [66]). There is evidence that face individuation changes with experience, as adult face-selective regions show larger face individuation responses compared to children [66], and adults exhibit larger FIE compared to children as measured with FPVS-EEG [24]. Moreover, children and adults show larger discrimination responses to own- compared to other-age faces in some face-selective regions [64,66]. However, the difference between children and adults could reflect changes toward later stages of maturation and thus does not provide direct evidence that experience exclusively acquired in adulthood influences the face identity recognition system. Thus, our findings add to this literature, suggesting that the functional architecture underlying FIR remains plastic long after maturation. Whether the plasticity reflects the recruitment of more neural sources to process inverted faces—and whether these would be recruited exclusively from face-selective neurons—and/or stronger sensitivity within neuronal sources for inverted faces, remain unknown and are topics for future studies. Moreover, we found that the training effect was present one day after the training stopped, but it is currently unknown if it would persist for a long time when the participants no longer experience inverted faces.

It is thought that the larger inversion effect for faces compared to non-face objects [7] reflects processing of upright faces as an integrated whole entity of diagnostic face features (eyes, nose, mouth), rather than a more part-based collection of features. This “holistic/configural” view is supported by a large body of evidence showing that inversion reduces well-known behavioral markers of holistic processing, including the “whole-part advantage” [70,71] and the “composite face effect” [72], as well as sensitivity to spatial relationships between (distant) facial features [19,73,74,75,76]. To account for these phenomena, it has been proposed that inversion shrinks the “perceptual field” (or functional field of vision) when dealing with face identity recognition [77], explaining a change in relative performance for gaze-contingent window and mask conditions for upright and inverted faces [78]. In line with this proposal, a recent fMRI study found that face-selective regions exhibited larger population receptive fields (pRFs) and visual field coverage for upright compared to inverted faces, where the visual field coverage of upright faces included the eye and the mouth regions, while that of inverted faces missed the mouth [79]. In the current study, it is possible that the inverted face training expanded the perceptual field for inverted faces, thereby increasing the discrimination ability of inverted faces, (e.g., [80]), without changing the optimal fixation point away from the eyes [2]. Future work could examine if inverted face discrimination training broadens the perceptual field coverage for inverted faces, as measured with behavioral measures of holistic processing or fMRI pRF mapping. A possible mechanism for broadening visual field coverage could be strengthening the functional connectivity between face-selective regions and retinotopic regions with upper visual field bias—increasing the retinotopic convergence in face-selective regions—which could be examined using functional connectivity analyses; (see also [81]).

Note that there is evidence that experience can influence both part-based and holistic processing. On one hand, a behavioral study found that inverted face training improved part-based learning of inverted faces [42]. However, this effect could reflect that training was conducted with a small set of images that were also used in the test phase (i.e., no generalization to novel faces was tested and a small set of face images were trained thereby potentially allowing for learning image cues). On the other hand, a wide range of evidence indicates that experience can influence holistic processing. In the face domain, preschool teachers and maternity ward nurses, who benefit from prolonged visual experience with child and newborn faces, respectively, display similar hallmarks of holistic processing when presented with adult and child/infant faces, while novices show stronger holistic effects for own-age faces [37,38,82]. In the non-face domain, behavioral markers of holistic perception have been reported for people with extensive real-world experience discrimination objects within a domain (i.e., domain experts), including car experts recognizing cars [83], chess experts recognizing chess-board configurations [84], bird watchers recognizing birds [85], and in laboratory-trained experts recognizing artificial objects [86]. Moreover, it has been shown that discrimination training with novel objects is more effective when the stimuli to be learned have face-like configurations [87]. Overall, while this indicates that inverted faces training could, in principle, lead to more holistic processing of inverted faces, this would need to be verified by future studies.

In summary, we revealed that extensive inverted face discrimination training in adulthood influenced both behavioral and neural inverted face discrimination. The behavioral findings replicate and support previous behavioral evidence [41], and crucially, the neural findings show novel evidence pinpointing the training effects to cortical perceptual face identity recognition processes in the right occipito-temporal cortex. This casts new light on the plasticity of the FIR system: while it is widely accepted that children’s cortical face processes are highly malleable [88,89], our findings show that also adult face processing can be highly malleable to visual experience. Future work should directly compare the malleability in children and adults using tasks where both groups show malleability, such as the tasks of the current study. For example, it could be that the FIE would be entirely abolished in children due to higher plasticity. Thus, overall, our findings substantially strengthen the notion that the mature FIR system is highly plastic and opens up for a range of new questions regarding why and how this system changes with experience in our everyday adult lives.

## Figures and Tables

**Table 1:** Test and training program.

Pre-test, day 1, week 1 (Friday)					
**1**	Forced-choice matching task Set 0 (32 faces)				
**Learning session**					
**Session 1, day 4, week 2 (Monday)**			**Estimated time**		
**1**	Exposure/learning task Set 1 (10 faces)		±	10	min
**2**	Naming task Set 1		±	75	min
**3**	Forced-choice matching task		±	25	min
**Session 2, day 5, week 2 (Tuesday)**					
**1**	Exposure/learning task Set 1		±	10	min
**2**	Naming task Set 1		±	50	min
**3**	Old/New task Set 1		±	5	min
**4**	Exposure/learning task Set 2 (10 faces)		±	10	min
**5**	Naming task Set 2		±	20	min
**6**	Naming task Set 1 + Set 2		±	15	min
**Session 3, day 6, week 2 (Wednesday)**					
**1**	Exposure/learning task Set 1		±	10	min
**2**	Exposure/learning task Set 2		±	10	min
**3**	Naming task Set1 + Set 2		±	60	min
**4**	Forced-choice matching task		±	20	min
**5**	Naming task Set 1 + Set 2		±	15	min
**Session 4, day 7, week 2 (Thursday)**					
**1**	Naming task Set 1 + Set 2		±	60	min
**2**	Exposure/learning task Set 1		±	10	min
**3**	Exposure/learning task Set 2		±	10	min
**4**	Old/New task Set 1 + Set 2		±	10	min
**5**	Naming task Set 1 + Set 2	(two successes required)	±	15	min
**Session 5, day 8, week 2 (Friday)**					
**1**	Old/New task Set 1 + Set 2		±	10	min
**2**	Naming task Set 1 + Set 2		±	30	min
**3**	Exposure/learning task Set 3		±	10	min
**4**	Naming task Set 3 (10 faces)		±	15	min
**5**	Forced-choice matching task		±	15	min
**6**	Naming task Set 3		±	25	min
**7**	Naming task Set 1 + Set 2 + Set 3		±	15	min
**Session 6, day 11, week 3 (Monday)**					
**1**	Exposure/learning task Set 3		±	10	min
**2**	Naming task Set 1 + Set 2 + Set 3		±	40	min
**3**	Old/New task Set 1 + Set 2 + Set 3		±	15	min
**4**	Forced-choice matching task		±	15	min
**5**	Naming task Set 1 + Set 2 + Set 3	(two successes required)	±	40	min
**Session 7, day 12, week 3 (Tuesday)**					
**1**	Old/New task Set 1 + Set 2 + Set 3		±	15	min
**2**	Forced-choice matching task		±	15	min
**3**	Naming task Set 1 + Set 2 + Set 3	(two successes required)	±	40	min
**4**	Old/New task Set 1 + Set 2 + Set 3		±	15	min
**5**	Naming task Set 1 + Set 2 + Set 3	(two successes required)	±	40	min
**Session 8, day 13, week 3 (Wednesday)**					
**1**	Forced-choice matching task		±	15	min
**2**	Naming task Set 1 + Set 2 + Set 3	(two successes required)	±	40	min
**3**	Old/New task Set 1 + Set 2 + Set 3		±	15	min
**4**	Naming task Set 1 + Set 2 + Set 3		±	40	min
**Post-test, day 14, week 3 (Thursday)**					
**1**	Forced-choice matching task (Set 4)				

## Data Availability

Data are available at the Open Science Framework (https://osf.io/8y6hx/), accessed on 1 December 2023.
